# Management of Upper Respiratory Tract Infections by Different Medical Practices, Including Homeopathy, and Consumption of Antibiotics in Primary Care: The EPI3 Cohort Study in France 2007–2008

**DOI:** 10.1371/journal.pone.0089990

**Published:** 2014-03-19

**Authors:** Lamiae Grimaldi-Bensouda, Bernard Bégaud, Michel Rossignol, Bernard Avouac, France Lert, Frederic Rouillon, Jacques Bénichou, Jacques Massol, Gerard Duru, Anne-Marie Magnier, Lucien Abenhaim, Didier Guillemot

**Affiliations:** 1 LA-SER, Paris, France; 2 Pharmacoepidemiology and Infectious Diseases Research Group, Pasteur Institute, Paris, France; 3 INSERM U657, University of Bordeaux Segalen, U657, Bordeaux, France; 4 Department of Epidemiology, Biostatistics and Occupational Health, McGill University, Montreal, Canada; 5 LA-SER Center for Risk Research, Montreal, Canada; 6 INSERM U1018, Center for Epidemiology and Population Health, Villejuif, France; 7 Sainte-Anne Hospital, University of Paris V René Descartes, Paris, France; 8 INSERM U657, Pharmacoepidemiology and evaluation of the impact of health products on human health, Department of Pharmacology, University Victor Segalen Bordeaux 2, Tondu Hospital – Case 41, Bordeaux, France; 9 Department of Biostatistics, University Hospital of Rouen, Rouen, France; 10 Faculty of Medicine, University of Franche Comté, Besançon, France; 11 Cyklad Group, Rillieux la Pape, France; 12 Faculty of Medicine, University Pierre and Marie Curie, Paris, France; 13 Department of Epidemiology, London School of Hygiene and Tropical Medicine, London, United Kingdom; 14 LA-SER Europe Limited, London, United Kingdom; 15 Pasteur Institute, Paris, France; 16 Faculty of Medicine, University of Paris-Ile de France Ouest, Guyancourt, Paris, France; CUNY, United States of America

## Abstract

**Background:**

Prescribing of antibiotics for upper respiratory tract infections (URTI) varies substantially in primary care.

**Objectives:**

To describe and compare antibiotic and antipyretic/anti-inflammatory drugs use, URTI symptoms' resolution and occurrence of potentially-associated infections in patients seeking care from general practitioners (GPs) who exclusively prescribe conventional medications (GP-CM), regularly prescribe homeopathy within a mixed practice (GP-Mx), or are certified homeopathic GPs (GP-Ho).

**Method:**

The EPI3 survey was a nationwide population-based study of a representative sample of 825 GPs and their patients in France (2007–2008). GP recruitment was stratified by self-declared homeopathic prescribing preferences. Adults and children with confirmed URTI were asked to participate in a standardized telephone interview at inclusion, one-, three- and twelve-month follow up. Study outcomes included medication consumption, URTI symptoms' resolution and potentially-associated infections (sinusitis or otitis media/externa) as reported by patients. Analyses included calibration to account for non-respondents and groups were compared using multivate analyses adjusting for baseline differences with a propensity score.

**Results:**

518 adults and children with URTI (79.3% rhinopharyngitis) were included (36.9% response rate comparable between groups). As opposed to GP-CM patients, patients in the GP-Ho group showed significantly lower consumption of antibiotics (Odds ratio (OR) = 0.43, 95% confidence interval (CI): 0.27–0.68) and antipyretic/anti-inflammatory drugs (OR = 0.54, 95% CI: 0.38–0.76) with similar evolution in related symptoms (OR = 1.16, 95% CI: 0.64–2.10). An excess of potentially-associated infections (OR = 1.70, 95% CI: 0.90–3.20) was observed in the GP-Ho group (not statistically significant). No difference was found between GP-CM and GP-Mx patients.

**Conclusion:**

Patients who chose to consult GPs certified in homeopathy used less antibiotics and antipyretic/anti-inflammatory drugs for URTI than those seen by GPs prescribing conventional medications. No difference was observed in patients consulting GPs within mixed-practice. A non-statistically significant excess was estimated through modelling for associated infections in the GP-Ho group and needs to be further studied.

## Introduction

High antibiotic consumption is said to be associated with the emergence and dissemination of multi-resistant bacteria in the community [1]. Demands and expectations for antibiotics in common upper respiratory tract infections [URTI]) are important drivers of antibiotic overprescribing in primary care [2]. Many countries have initiated programs targeted at physicians and the general public to reduce antibiotic prescribing [3]. Most evaluated programs have recorded some success even though the effect on resistance to antimicrobial drugs, and particularly on dissemination of antibiotic-resistant pneumococci, remains uncertain [4]. Substantial heterogeneity in antibiotic prescribing among French GPs has been observed [5]. Despite the modest decrease in ambulatory antibiotic prescribing for respiratory tract infections between 2001 and 2009, France remains a country with one of the highest antibiotic consumption rates in Europe [6], [7].

While there is evidence that homeopathy has little effect on URTI or flu-like symptoms [8], [9], its potential for reducing antibiotic consumption has been proposed [10]. In France, homeopathic medicines are partially reimbursed by the National Health Insurance and are prescribed exclusively by a physician. Besides, patients must choose a ‘treating physician’, who will be responsible for follow-up and referral to specialists. This treating physician may be a physician specializing in homeopathy. This context provided a unique opportunity to observe homeopathic prescribing practices in the management of patients with URTI in primary care.

The objectives of this one-year population-based cohort study was to describe and compare antibiotic and antipyretic/anti-inflammatory drugs use, resolution of URTI symptoms and occurrence of potentially associated infections in patients who seek care for URTI from general practitioners (GPs) showing different prescribing preferences for homeopathy: strictly prescribers of conventional medications reluctant to prescribe homeopathic medicines (GP-CM), regular prescribers of homeopathic medicines in an otherwise conventional medical practice (“mixed prescribing” or GP-Mx), and certified homeopathic GPs (GP-Ho), who also prescribe conventional medications.

## Methods

### Study design and population

The EPI3 survey was a nationwide survey of primary care practice conducted in a representative sample of GPs from across France and their patients between 2007 and 2008 [11]. The sample was drawn using a two-stage sampling process. First, a random sample of GPs was drawn from the French national directory of physicians in primary care. Sampling of GPs was stratified according to self-declaration of prescribing preferences obtained by telephone at the time of recruitment and categorized into three groups: strictly prescribers of conventional medications (GP-CM) who declared never using homeopathy, or only at the patient's request; regular prescribers of homeopathic medicines within a mixed prescribing practice (GP-Mx); and certified homeopathic GPs (GP-Ho). Second, a one-day survey of all patients attending the medical practice of each participating GP was conducted where a trained research assistant surveyed all patients in the waiting room. For this cohort study, the first 5 (minimum) to 15 (maximum) consenting adult patients and guardians of children were invited to participate if (a) the attending physician was declared by patients as their regular physician (designated “treating physician” according to French regulation) and (b) one of the clinical diagnosis declared by the physician at that visit included one of the following ICD-9 (International Classification of Diseases 9^th^ revision) [12] codes: acute nasopharyngitis [common cold] (code 460), acute upper respiratory infections of multiple or unspecified sites (code 465); acute bronchitis and bronchiolitis or bronchitis, not otherwise specified (codes 466 and 490, respectively), acute pharyngitis (code 462) and acute laryngitis and tracheitis (code 464).

### Data collection

At inclusion, GPs completed a medical questionnaire for each patient included in the cohort with the main reason diagnosis, a standardized history of respiratory diagnoses in the previous year and of respiratory symptoms in the current episode of URTI, up to five other diagnoses (comorbidities) and all drugs prescribed that day. Diagnoses were coded according to the ICD-9 classification by a trained research assistant. All consenting patients completed a self-administered questionnaire at inclusion, in the waiting room, collecting information on lifestyle and history of medical consultations and hospitalizations in the previous year. The follow-up telephone interview at one month included the inventory of URTI symptoms obtained via patients' self-assessment of changes in those symptoms from baseline (cleared, much improved, slightly improved, no change or worsened). Interviews at one, three and twelve months spanned the patient's history since the previous interview with regard to the occurrence of infections associated with the URTI, defined as patients' self-report of a diagnosis (with or without treatment) of otitis (media or externa) and/or sinusitis, and any drug consumption (conventional and homeopathy). This calendar was used to aid patients' recall during the one-year follow-up. Drug consumption, whether prescribed or obtained over-the-counter or from the family pharmacy, was assessed using a standardized method named Progressive Assisted Backward Active Recall (PABAR) previously validated against medical prescriptions [13], [14]. Briefly, patients received at the time of their recruitment a booklet detailing the interview, including a list of commonly used drugs for URTIs, and were instructed to collect all their prescriptions. Trained interviewers helped patients recall past exposure to drugs, starting with recent history and progressing back in time to identify events at key dates. Drugs were automatically recorded using the anatomical therapeutic chemical classification index (ATC), 2009 revision. Particular emphasis was put on antibiotics [antibacterial for systemic use (class J01), neuraminidase inhibitors (class J05AH)], antipyretics [acetaminophen (class N02BE01), acetylsalicylic acid (class N02BA01)] and non-steroidal anti-inflammatory drugs (NSAIDs) [ibuprofen (class M01AE01)], as well as homeopathic drugs commonly used in URTI. Patients were asked to specifically report consumption of any drug from a list of 41 products after they had spontaneously reported all drugs used. Patients also reported the occurrence of diagnoses (with or without treatment) of otitis (media or externa) and/or sinusitis. Those two diagnoses were used as proxies for potentially associated infections.

### Study outcomes

Four outcomes were evaluated as yes/no responses (Table 1). Consumption of antibiotics and antipyretic/anti-inflammatory drugs for URTI was defined at each interview interval as the proportion of patients who declared using at least one drug (since the previous interview) from any of the ATC classes listed above. Utilization of antibiotics and antipyretic/anti-inflammatory drugs was then defined as at least one utilization for URTI at any of the one, three or twelve-month interviews. Resolution of the URTI was defined following either patients' self-report of complete resolution or significant improvement of baseline symptoms at the one-month interview. Infections potentially associated to the URTI were defined following patients' self-report of at least one declaration of a diagnosis of otitis media, otitis externa or sinusitis (with or without treatment) at any of the one, three or twelve-month interviews.

**Table 1:** Comparison of drug utilization, resolution of URTI symptoms and occurrence of potentially associated infections among types of medical practice§.

	Odds ratio¥ [95% confidence interval] (number of events)		
	GP-CM (N = 165)	GP-Mx (N = 203)	GP-Ho (N = 150)
**Drug utilization**			
**Antibiotic use** (Used at least once for URTI in 12 months)	1.00 (75)	1.07 [0.79–1.46] (91)	**0.43 [0.27–0.68]** (27)
**Antipyretic/anti-inflammatory drug use** (Used at least once for URTI in 12 months)	1.00 (106)	1.23 [0.91–1.65] (126)	**0.54 [0.38–0.76]** (61)
**Resolution of the URTI Symptoms resolved or greatly improved** (Self-declaration at 1 month)	1.00 (110)	1.10 [0.63–1.91] (141)	1.16 [0.64–2.10] (104)
**Potentially associated infections** (At least one declaration of otitis/sinusitis in 12 months)	1.00 (24)	0.88 [0.46–1.71] (23)	1.70 [0.90–3.20] (32)

§ Type of medical practice according to physicians' prescribing preferences: GP-CM, conventional medicine used as the category of reference; GP-Mx, mixed prescribing practice (conventional and homeopathic); GP-Ho, registered homeopathic physicians.

¥ Odds ratios and 95% confidence intervals obtained by logistic regression using GEE models adjusted for all variables in Tables 2 and 3.

### Statistical analysis

Participants and nonparticipants in the cohort study were compared using information collected from all surveyed patients at baseline. Characteristics of patients not participating in the survey (gender, age and main reasons for consultation) were used to calibrate the final sample using a method known as the CALMAR procedure [15]. In this method, differences between participants and nonparticipants were compensated by attributing a specific weight to each patient in the analysis, inversely proportional to the participation rate of similar patients at baseline among all patients surveyed. Differences at baseline between GP-CM, GP-Mx and GP-Ho groups were estimated using multivariate logistic regression analyses. A propensity score was computed for each participant in the study indicating their probability of belonging to either GP-Mx or GP-Ho groups compared to the GP-CM group according to all variables listed in Table 2. The score was intended to adjust for confounding by differences between the groups in all subsequent analyses.

**Table 2:** Baseline characteristics of patients with upper respiratory tract infections (URTI) by type of medical practice§.

	Total	GP-CM	GP-Mx	GP-Ho	Nonparticipating patients
	N = 518 (%)	N = 165 (%)	N = 203 (%)	N = 150 (%)	N = 884 (%)
Female gender	59.8	58.2	58.6	63.3*	54.2¥
Age (years)					
0–19	41.9	38.2	43.3	44.0	40.5
20–49	33.6	41.2	29.1	31.3	39.7
50+	24.5	20.6	27.6	24.7	19.8¥
High school degree completed (adults only)	57.1	55.7	50.8	65.7*	53.4¥
Body Mass Index (adults only)					
<25	60.6	65.0	51.3	68.3	61.9
25+	39.4	35.0	48.7	31.7	38.1
Smoking (adults only)					
Non smoker	47.3	36.4	50.2	55.3*	39.6
Former smoker (>1 year)	23.0	27.9	20.7	20.7	21.4
Current smoker or recent smoker (<1 year)	29.7	35.7	29.1	24.0	39.0¥
Physical activity ≥30 minutes per day (adults only)	28.1	33.5	25.7	25.5	29.7

§ Type of medical practice according to physicians' prescribing preferences: GP-CM, conventional medicine; GP-Mx, mixed practice (conventional and homeopathic); GP-Ho, registered homeopathic physicians.

¥ Differences compared to participants statistically significant (p<0.05).

*Differences compared to the GP-CM group statistically significant (p<0.05).

The three groups were compared for the four binary outcomes using the GP-CM group as the reference group using logistic regression analyses adjusted for baseline characteristics (propensity score) and the number of URTI symptoms at baseline (as listed in Table 3). Clustering effects resulting from recruiting several patients consulting the same GP and autocorrelation between responses to the four consecutive interviews were controlled using Generalized Estimating Equations (GEE) in the multivariate models. Sample size was estimated for the EPI3 survey as a whole so as to provide accurate estimates of prevalence (at ±2%) for each group of diagnoses seen in primary care, including URTI. All analyses were performed using SAS version 9.1 (SAS Institute, Inc., Cary, North Carolina).

**Table 3:** Baseline clinical characteristics of upper respiratory tract infections (URTI) by type of medical practice§.

	Total	GP-CM	GP-Mx	GP-Ho
	N = 518 (%)	N = 165 (%)	N = 203 (%)	N = 150 (%)
URTI symptoms at inclusion				
Fever ≥38.5°C	51.0	53.3	59.1	37.3*
Rhinorrhea	72.4	75.2	71.4	70.7
Nasal obstruction	57.3	63.0	56.2	52.7*
Cough	71.2	76.4	72.4	64.0*
Shortness of breath	30.1	27.9	36.5	24.0
Dysphagia	21.8	22.4	23.6	18.7
Stomatitis	4.1	3.6	5.9	2.0
Diagnoses/syndrome at inclusion				
Rhinopharyngitis	73.9	75.2	70.9	76.7
Bronchitis	28.0	29.1	31.5	22.0
Bronchiolitis	5.2	6.1	5.4	4.0
Flu-like symptoms	12.7	10.9	14.8	12.0
Viral angina (negative Strep-A rapid test)	8.7	9.1	9.9	6.7
Comorbidities				
Gastroenteritis	3.5	0.6	5.9	3.3
URTI in previous 12 months	67.2	64.2	68.5	68.7
Hospitalization in previous 12 months(all causes)	14.1	16.0	14.4	11.5
Prescriptions on day of inclusion (at least one in each category as follows)				
Antibiotic	32.6	39.4	41.4	13.3*
Antipyretic/anti-inflammatory drugs	40.5	52.1	45.8	20.7*
Homeopathic drug for URTI	21.6	0.6	9.4	61.3*

§ Type of medical practice according to physicians' prescribing preferences: GP-CM, conventional medicine; GP-Mx, mixed prescribing practice (conventional and homeopathic); GP-Ho, registered homeopathic physicians.

*Difference statistically significant (p<0.05).

### Ethics Statement

The study was approved by the French National Data-Protection Commission (CNIL) and the French National Medical Council (CNOM). In accordance with CNIL regulation, written consent was obtained from each participating adult patient and from one of the parents accompanying each participating child. Participating physicians received compensation fees for their participation but not patients.

## Results

The general EPI3 health survey included 825 GPs and 8,559 participants (ten patients per GP on average, minimum 5, maximum 15). Of the latter, 1,402 children and adults fulfilled the specific inclusion criteria for the URTI cohort, of which 699 (49.9%) agreed to participate with 518 (36.9%) responding to all three follow-up interviews and therefore included in the analysis. Participants were slightly more often females (59.8%) compared to nonparticipants (54.2%), more likely to belong to the 50+ years age group (24.5% and 19.8%, respectively), to have completed high school education (57.1% and 53.4%), and less likely to be a current smoker (29.7% and 39.0%, respectively), all differences statistically significant (Table 2). Of participants under 20 years of age (41.9%), two thirds (28.1%) were 6 years old or younger. Among participants, patients who consulted a GP-Ho were more often non-smoking females who completed high school education compared to the GP-CM group, differences that were statistically significant after taking into account all other factors, and were similar otherwise (Table 2). Patients in the GP-Mx group were comparable to the GP-CM group.

With regard to types of URTI at baseline, there were little differences between the three groups. The most commonly reported was rhinopharyngitis (73.9%), followed with bronchitis (28.0%), flu-like symptoms (12.7%), Strep-A negative viral angina (8.7%) and bronchiolitis (5.2%) (Table 3). For symptoms reported by patients, those who consulted a GP-Ho were less likely to have fever (37.3%), nasal obstruction (52.7%) and cough (64.0%) than those consulting a GP-CM (53.3%, 63.0% and 76.4%, respectively; all three statistically significant). Patients in the GP-Mx were comparable to those in the GP-CM group.

Prescribing preferences of physicians in the three groups were confirmed at baseline by their respective prescribing rates of homeopathic drugs, which were 0.6%, 9.4% and 61.3%, respectively, in the GP-CM, GP-Mx and GP-Ho groups (Table 3). Conversely, antibiotic and antipyretic/anti-inflammatory drug prescriptions, which were relatively comparable between the GP-CM and the GP-Mx groups with rates above 40%, were much lower in the GP-Ho group with rates at or below 20%.

Table 1 shows the multivariate analyses on drug utilization, resolution of the URTI symptoms and occurrence of potentially associated infections. For antibiotic and antipyretic/anti-inflammatory drug utilization, the lower consumption observed at baseline in the GP-Ho group was maintained over the twelve-month follow-up with an adjusted probability of patients' reporting significantly lower than the GP-CM group both for antibiotics (OR = 0.43; 95% CI: 0.27–0.68) and for antipyretic/anti-inflammatory drugs (OR = 0.54; 95% CI: 0.38–0.76). There was no difference in drug utilization between the groups GP-Mx and GP-CM.

For resolution of the URTI symptoms at one-month follow-up, patients declared similar improvement in the three groups (77.0%, 81.1% and 77.7% respectively in the GP-CM, GP-Mx and GP-Ho groups) with no statistically significant differences in multivariate analyses adjusting for baseline characteristics (OR = 1.16; 95% CI: 0.64–2.10). On the other hand, there was a slightly higher rate of potentially associated infections (otitis media/externa and sinusitis) reported by patients in the GP-Ho group over twelve months follow-up (17.7%) compared to the GP-CM group (16.9%), a difference that was not statistically significant (OR = 1.71; 95% CI: 0.90–3.20). Results on symptoms resolution and occurrence of potentially associated infections exhibited wide confidence intervals.

## Discussion

This population-based prospective cohort study described and compared clinical management and evolution of patients consulting for URTI between three groups of physicians with different levels of prescribing preferences for homeopathy. At baseline, patients who chose to be seen by GP-Ho for URTI declared to have used half the amount of antibiotics and antipyretic/anti-inflammatory drugs compared to patients seen by conventional medicine practitioners. This lower consumption of conventional medications in the GP-Ho group was sustained over the 12-month follow-up. At the same time, no difference in the resolution of the URTI symptoms was observed between groups but confidence intervals were wide indicating lack of statistical power for that outcome. Similarly, the excess rate of potentially associated infections observed in the GP-Ho group, although non-statistically significant, cannot be ruled out. No difference was seen in patients from the GP-Mx group, which was comparable to the GP-CM group on all outcomes.

Previous observational studies conducted in several countries have shown an antibiotic-sparing effect resulting from management by GPs using homeopathy without increase in complication rates of URTI [10], [16]. Patients' education, including appropriate indication for antibiotic use, infection prognosis, and alternative treatment recommendations, may contribute to lower patients' expectations toward antibiotics while improving satisfaction [2]. This has been described in France during the 2009–2010 influenza season [17]. Authors have pointed out the difficulty of sorting out patients' expectations/motivation and homeopathic care itself, including their providers [18], [19].

The rise in bacterial resistance to antibiotics is widely recognized as a major threat to public health [6], [20]. Antibiotic prescribing for URTI varies widely within and across countries [21], [22] suggesting that further control of antibiotic prescribing is possible. Many countries have implemented policies aimed at reducing inappropriate prescribing of antimicrobials in primary care [6], [22]. In that context, our results are not unexpected and can contribute to reinforce the motivation of decision makers to pursue these policies [23]–[25].

Our results could be explained in part by the different characteristics of patients seen by GPs who practice homeopathy and by the lower rate of fever, nasal obstruction and cough in the GP-Ho group at baseline compared to the two other groups. Adjustment by severity of URTI and other potential confounders did not alter the results but residual confounding cannot be excluded. As for our observation of a small non-statistically significant excess in the occurrence of potentially associated infections in the GP-Ho group, it may be due to chance or to a lack of protection against these infections. The latter instance cannot be ruled out as the study lacked statistical power to distinguish between the two interpretations.

### Study limitations

The participation rate in this URTI cohort study was only 36.9% of eligible patients, which is comparatively equivalent to what is seen in general health surveys where patients are asked to participate in a long follow-up [26]. Given that this study was appended to a general population health survey, contributed at reducing the risk of selection bias of physicians and patients. The overall prevalence of URTI in this survey (12.4%; 95% CI: 11.5–13.2) was compatible with statistics on GP consultations in France [11]. Precautions taken to calibrate the final sample so as to ensure representatively of the eligible population contributed at reducing sampling bias but without ruling it out entirely. The results may also be subject to residual confounding because the propensity score may have not accounted for all the differences between patients who seek treatment from different types of physicians.

Another potential limitation is related to the nature of URTI diagnoses that have not been validated against a disease management guideline. No such attempt was made to preserve the authenticity of primary care practice in real life. This is partially why diagnoses of bronchitis and bronchiolitis were included in this cohort as they may represent co-occurrences of URTI. The standardized collection of symptoms allowed a partial control for severity of URTI at inclusion. Two conditions, sinusitis and otitis, were studied as proxies for the occurrence of infections potentially associated to the URTI. Diagnoses were obtained from patients' self-declaration over the telephone and should not be interpreted strictly. It is not known whether they represent true complications or URTI and/or represent associated infections as a result of no antibiotic treatment. This should be studied, particularly in view of the apparent excess of infections observed in the GP-Ho group. However, the lack of diagnostic confirmation should not bias the comparison between the groups but may bias the results toward the null and thus reducing the statistical significance of the observation. In view of the different characteristics of patients in the GP-Ho group at inclusion, the lower frequency of symptoms reported that group might be explained by a lower threshold of these patients to consult a physician rather than a true difference in the diagnoses makeup of the group.

Finally, results on resolution of URTI symptoms were underpowered to show non-inferiority between groups as illustrated by the wide confidence intervals. The estimates however were close to unity in both GP-Mx and GP-Ho groups, indicating similarity for self-declaration of symptoms resolution at one month between patients from both groups. Sample size was sufficient to show an Odds ratio superior to 1.22 (or inferior to 0.82) for the main outcomes. Strengths of this study included the length of follow-up and the quality of the data which combined medical and patient information collected from physicians and patients. Drug consumption was obtained from patients interviews using a validated approach [13], [14] that allowed the identification of prescription drugs as well as those obtained over-the-counter or from the family pharmacy, the latter being known to be an important source of self-treatment for URTI [27].

In conclusion, this cohort study showed that patients with URTI who choose to consult homeopathy-certified GPs in primary care, had a lower consumption of antibiotics and antipyretic/anti-inflammatory drugs as compared to patients seen by physicians who use conventional medicine. This difference may be due to specific attributes of either physicians or patients but also interactions between the two. No difference was observed for patients consulting GPs with mixed prescribing habits. The non-significant excess of potentially associated infections in the GP-Ho group esteemed through modelling may be due to chance alone or driven by less use antibiotics. Further studies are needed to clarify this effect. Other large studies are needed to establish the long-term consequences of different prescribing practices in primary care.
